# Spectroscopic Studies of the Modulation of Metabolic Activity of HBE Cells by Doxorubicin Hydrochloride-Loaded SW480-Derived Exosomal Nanocarriers

**DOI:** 10.3390/cancers18030379

**Published:** 2026-01-26

**Authors:** Hubert Grel, Katarzyna Ratajczak, Weronika Daria Krahel, Anna Słońska, Joanna Cymerys, Marcin Wisniewski, Magdalena Stobiecka

**Affiliations:** 1 Department of Physics and Biophysics, Institute of Biology, Warsaw University of Life Sciences, 159 Nowoursynowska Street, 02776 Warsaw, Poland; hubert_grel@sggw.edu.pl (H.G.); katarzyna_ratajczak@sggw.edu.pl (K.R.); 2 Department of Preclinical Sciences, Institute of Veterinary Medicine, Warsaw University of Life Sciences, 8 Ciszewskiego Street, 02786 Warsaw, Poland; weronika_krahel@sggw.edu.pl (W.D.K.); anna_slonska@sggw.edu.pl (A.S.); joanna_cymerys@sggw.edu.pl (J.C.); marcin_wisniewski@sggw.edu.pl (M.W.)

**Keywords:** exosomes, doxorubicin, SW480 cells, colorectal cancer, fluorescence spectroscopy, UV-Vis spectroscopy, nanocarriers, HBE cells

## Abstract

Background: The high efficiency of chemotherapy in cancer treatment is due to the highly effective killing of cancer cells by the applied anticancer drugs. However, this requires a systemic distribution of anticancer drugs which may lead to adverse effects. Objectives: Therefore, in this work, we have examined the utility of a model targeted anticancer drug delivery based on exosomal nanocarriers. The aim of this study was to investigate the feasibility of doxorubicin anticancer drug loading into the exosomes, secreted by the human colon cancer cells (SW480), and to determine the effectiveness of this process. Methods: Exosome isolation was confirmed by molecular biology techniques, including capillary-based Simple Western immunoassay, as well as by nanoparticle tracking analysis and transmission electron microscopy imaging. The loading of exosomes with drugs was monitored using fluorescence spectroscopy and the standard addition method. Furthermore, the cellular uptake of doxorubicin-loaded exosomes was analyzed using laser scanning confocal microscopy. Results: It has been shown that 15–17% of doxorubicin could be loaded into the exosomal nanocarriers using the electroporation process. The modulation of metabolic activity of human bronchial epithelial (HBE) cells was achieved by stimulating these cells with doxorubicin encapsulated in exosomes, as evidenced by monitoring with the UV-Vis Alamar Blue assay. Conclusions: The obtained results seem to be particularly interesting, considering the high internalization efficiency of SW480-derived exosomes to HBE cells in comparison with the other evaluated cell lines (SW480, CCD 841 CoN, A549).

## Simple Summary

Since colorectal cancer (CRC) is one of the most common and terminal cases of cancer in the world, effective methods of targeted anticancer drug delivery systems and investigation of the processes of cancer metastasis are of high importance. In this work, we propose a novel platform for safe and efficient drug delivery based on exosome nanocarriers. The investigations performed have shown successful isolation of exosomes from the human colon cancer cell line SW480. To understand CRC progression, we investigated the modulation of metabolic activity of the human bronchial epithelial cell line 16HBE14σ by doxorubicin-loaded exosomes using UV-Vis Alamar Blue assay. The results obtained corroborate the high potential of the exosomes as the novel drug delivery system and enable better understanding of the role of SW480-derived exosomes on taming the CRC spread.

## 1. Introduction

Colorectal cancer (CRC) is one of the most common and fatal cases of cancer in the world [1,2]. Therefore, a better understanding of the mechanisms of its progression and metastatic dissemination is urgently needed for CRC diagnosis. The effective methods of targeted drug delivery systems and cancer treatment are also crucial issues for CRC therapy. The best way of treating cancers is still a systemic method of chemotherapy [3]. However, because of the drugs’ traveling throughout the body, the normal, healthy cells, and especially the rapidly dividing cells, are prone to damage by highly aggressive chemotherapeutic agents, which also cause many side effects, including hair loss, infection, anemia, fatigue, and others. It was also shown that doxorubicin, which is an anthracycline chemotherapeutic drug commonly used to treat many types of cancer, caused cardiotoxicity [4], mitochondria, and blood vessel damage and induced autophagy [5]. One way to mitigate the negative effects of anticancer drugs on healthy cells and reduce its systemic toxicity is to deliver the drugs directly to the cancer cells by incorporating them into micelles [6], liposomes [7,8], exosomes [9,10], nanoparticles [11,12], or other nanostructures [13,14,15].

Exosomes (EXOs) are naturally occurring extracellular nanovesicles surrounded by a lipid bilayer membrane. Their size varies from 40 to 160 nm [16,17]. Exosomes are secreted by most eukaryotic cells. They carry natural cargoes such as proteins, lipids, and nucleic acids, including miRNA, siRNA, DNA, and mRNA [18,19,20], that are characteristic for parental cells; therefore, they are promising biomarkers for different disease detection with diagnostic potential and belong to one of the liquid biopsy components [21]. The discovery of exosomes derived from immune cells has resulted in advances in cancer immunotherapy through the enhancement of anticancer immune response [22].

The expression of various cellular and lipid adhesion molecules on the exosomes’ surfaces enables an increase in the targeting ability efficiency of exosomes and their cellular uptake, while the distribution of drugs by other delivery systems like liposomes can be less precise [23,24]. Furthermore, the exosomal lipid bilayer provides stability and bioavailability to the repertoire of bioactive molecules by protecting them from enzymatic degradation and enhances their delivery efficiency by reducing clearance rates. Liposome-based systems require modification of the membrane surface to achieve similar stability and improve their circulation half-lives [25]. The expression of endogenous or so-called self-markers allows the exosomes to avoid opsonization and consequently evade phagocytosis [26]. It was also shown that exosomes are also characterized by a little long-term accumulation in any organ or tissues with concurrent low systemic toxicity and immunogenicity in comparison with synthetic nanovesicles [24].

Due to their excellent features, including good biocompatibility, low immunogenicity, biodegradability, and stability, as well as the capacity to carry target-specific agents, the low-toxicity exosomes show strong potential for becoming excellent natural drug delivery nanocarriers [27,28,29], able to carry significant loads of drug cargoes [30,31,32]. Exosomes can also cross the blood–brain barrier (BBB), which is a major challenge in the treatment of neurological disorders [33]. It has recently been demonstrated that exosomes can serve as a delivery platform for cancer drugs, such as paclitaxel [34,35,36], doxorubicin [37,38,39], and Olaparib [40], as well as the anti-inflammatory agent curcumin [28,41].

Exosomes can also engage in programmed cell death and apoptosis by transferring pro- or anti-death molecules and pro-apoptotic factors, respectively, to neighboring cells [42].

Tumor-derived exosomes can also facilitate the spread of cancer, its progression, and induce cancerous malignant alterations in recipient cells including cell proliferation, extracellular matrix remodeling, cell migration, and angiogenesis [43,44]. It has also been shown that exosomes can promote an epithelial-to-mesenchymal transition (EMT) and enhance cells’ migratory activity [45].

Due to the growing interest in exosome research, owing to their properties and the possibility of utilization as a platform for drug delivery systems, the International Society for Extracellular Vesicles (ISEV) has released minimal information for studies of extracellular vesicles to advance rigor and transparency in studies and applications of extracellular vesicles. The first document was published in 2014, then in 2018 and 2023 (MISEV2014 [46], MISEV 2018 [47], and MISEV2023 [48], respectively).

Compared to other research on exosomes, this study aimed to investigate the role of exosomes in cancer progression and metastasis. We also focused on exploring how anticancer drugs incorporated into tumor-derived exosomes could influence healthy cells. Due to the particularly high metastasis of colon cancer to the respiratory system, we isolated exosomes from human colon cancer SW480 cells, loaded them with an anthracycline antibiotic doxorubicin (DOX), a model anticancer drug used in cancer therapy, and introduced them into healthy human bronchial epithelial HBE cells. We also investigated the feasibility of loading into exosomes and the utilization of exosomes as an efficient platform for doxorubicin drug encapsulation. The extracted exosomes from SW480 cancer cells were characterized using molecular biology techniques, including the automated capillary-based ProteinSimple immunoassay, nanoparticle tracking analysis (NTA), as well as transmission electron microscopy (TEM) imaging. The efficacy of the loading process was determined using fluorescence spectroscopy and, for the first time, the standard addition method. The obtained promising loading efficiency shows that the proposed encapsulation protocol can be successfully used by other scientists, also for other drugs. We also estimated the maximum number of DOX molecules that can fit in a single exosome nanocarrier. The uptake of exosomes loaded with doxorubicin (EXOs@DOX) in HBE cells was analyzed using laser scanning confocal microscopy. Next, the metabolic function and cellular health of human bronchial epithelial HBE cells after EXOs@DOX uptake were evaluated using UV-Vis Alamar Blue assay [49]. This paper demonstrates the potential of SW480-derived exosomes, their characterization, doxorubicin loading, and their inhibitory effect on the metabolic activity of HBE cells.

## 2. Materials and Methods

### 2.1. Chemicals

Fetal bovine serum (FBS), fetal bovine serum exosome-depleted (One Shot™), L-Glutamine, and Dulbecco’s modified Eagle’s Medium High Glucose (DMEM) were obtained from Gibco, Thermo Fisher Scientific, Waltham, MA, USA. Penicillin/streptomycin, doxorubicin hydrochloride (DOX), 3,3′-Dioctadecyloxacarbocyanine perchlorate (DiO), and 4′,6-Diamidine-2′-phenylindole dihydrochloride (DAPI) were purchased from Merck, Darmstadt, Germany. Phosphate-buffered saline (PBS) without magnesium and calcium ions (PBS pH 7.4 (1X)) was purchased from Gibco, Life Technologies, Loughborough, UK. Pierce Protease Inhibitor Tablets and Hoechst 33258 were obtained from Thermo Scientific, Waltham, MA, USA. ProLong™ Gold Antifade Mountant was purchased from Invitrogen™ Thermo Fisher Scientific, USA. All ready-to-use reagents and consumables used for automated capillary-based ProteinSimple immunoassay were obtained from the instrument manufacturer, Bio-Techne, Minneapolis, MN, USA.

All chemicals used for investigations were of analytical grade purity. Aqueous solutions were prepared with freshly deionized water with 18.2 MΩ × cm resistivity (Merck Millipore, Germany).

All concentrations of added reagents cited in this paper are final concentrations obtained after mixing. Curve fitting was performed using the Simplex algorithm.

### 2.2. Instrumentation

Fluorescence was recorded using Fluoroskan Ascent FL (ThermoFisher Scientific, USA). The excitation wavelength was set to *λ*_ex_ = 485 nm, and the emission wavelength was set to *λ*_em_ = 538 nm.

Absorbance was measured using Multiskan SkyHigh Microplate Spectrophotometer, (Thermo Fisher Scientific, USA).

The transmission electron microscopy (TEM) images of exosomes were recorded in the Electron Microscopy Platform of the Mossakowski Medical Research Centre of the Polish Academy of Sciences in Warsaw, Poland, using the transmission electron microscopy model JEM 1011 (JEOL) equipped with a model EDS INCA (Oxford) analyzer. The isolated exosomes by subsequent centrifugation and ultracentrifugation were diluted in buffer, and their suspension was placed onto a mesh copper grid with carbon-coated formvar film (200 mesh) for 10 min. Then, exosomes were stained with 2% aqueous uranyl acetate for 2 min. The accelerating voltage of the microscope during the observation was 80 kV [50].

Protein expression was analyzed using the fully automated capillary-based immunoassay ProteinSimple Jess system (Bio-Techne, USA).

The concentration of exosomes, as well as their mean size and size distribution, was determined using the nanoparticle tracking analysis (NTA) technique by the NanoSight NS300 system (Malvern Instruments Ltd., Worcestershire, UK) and the NTA 3.3 Dev Build 3.3.104 software. The exosomes were diluted with PBS and loaded into the sample chamber of NTA with a Laser Type: Blue488, settings: frames per second (fps) 25.0, temperature: 24 °C. The video (10 s) was recorded by adjusting the camera level to 16 with a detection threshold of 5.

The electroporation of exosomes was carried out in the electroporation cuvette with a 0.4 cm gap width in a Gene Pulse Electroporator (Bio-Rad, Hercules, CA, USA).

The cellular uptake of exosomes loaded with the drug doxorubicin was analyzed using the FluoView FV10i Confocal Laser Scanning Microscope (Olympus, Tokyo, Japan).

The electronic structure of doxorubicin (DOX) was calculated using the density functional theory (DFT) method with 6-311G* basis set embedded in Wavefunction Spartan ’14 (Irvine, CA, USA).

### 2.3. Cell Culture

The human colon cancer cell line SW480 purchased from ATCC (LGC Standards Sp. z o.o., Lomianki, Poland) was kindly obtained from Dr. Krazinski from the Department of Human Histology and Embryology of the University of Warmia and Mazury [51,52]. The human bronchial epithelial cell line 16HBE14σ (HBE) was kindly provided by Dr. Zajac from the Department of Physics and Biophysics of the Warsaw University of Life Sciences [53]. The human colon cancer cell line SW480, human bronchial epithelium HBE, and human lung carcinoma A549 cell lines were cultured in culture medium containing Dulbecco’s modified Eagle Medium High Glucose (DMEM) and CCD 841 ConN cell line in Eagle Minimum Essential Medium (EMEM) supplemented with 10% fetal bovine serum (*v*/*v*) (FBS, Gibco, Waltham, MA, USA), 1% penicillin/streptomycin (*v*/*v*), and 1% L-glutamine (*v*/*v*) and were maintained in a humidified atmosphere of 5% CO_2_ at 37 °C using a (Heracell™ 150i CO_2_ Incubator, Thermo Scientific, Waltham, MA, USA). The cell culture medium was replaced with a new medium every 2–3 days. After experiments, the used cells were collected and disposed appropriately according to safety protocols.

### 2.4. Exosomes Isolation

Exosomes were isolated from the supernatant of SW480 cells cultured for 48 h in DMEM media supplemented with 10% (*v*/*v*) exosome-depleted fetal bovine serum (Gibco OneShot, Thermo Fisher, USA), 1% penicillin/streptomycin, and 1% L-glutamine according to the procedure published earlier [54]. Briefly, cells (1 × 10^4^ cells/mL) were allowed to grow to 80% confluency. Exosomes were harvested from the cell culture supernatant by sequential centrifugation steps in an Eppendorf 5804R centrifuge. First, the supernatants collected in 50 mL Corning tubes (Sarstedt™, Nümbrecht, Germany) were centrifuged for 10 min at 300× *g* and 2000× *g* at 4 °C to remove alive cells and dead cells with the debris, respectively. Then, the supernatant was centrifuged for 30 min at 10,000× *g* at 4 °C to remove microvesicles and smaller cell debris. Next, the supernatant was ultracentrifuged in 26.3 mL tubes (PC Bottle, Beckman Coulter, Inc., Brea, CA, USA) for 120 min at 4 °C at a high speed of 120,000× *g* [55] in an ultracentrifuge (OPTIMA XPN-90, Beckman Coulter, Inc., Brea, CA, USA) to obtain the EXO pellet. Finally, the pelleted exosomes were resuspended in 75 µL PBS buffer, gently vortexed, and stored at 4 °C overnight. The next day, an exosomal pellet was used for the experiments or stored in 4 °C for a short time.

The concentration of exosome proteins was determined by Micro BCA Protein Assay Kit [56] (Thermo Fisher Scientific, USA) according to the manufacturer’s protocol. First, the serial dilution of the bovine serum albumin (BSA) solution was mixed with the three Micro BCA reagents A, B, and C and incubated for 2 h at 37 °C in duplicate to obtain the calibration curve. The BSA solutions absorbance was measured at 570 nm wavelength using a spectrophotometer (Multiskan SkyHigh Microplate Spectrophotometer, Thermo Fisher Scientific, USA). The exosome samples were prepared analogously. The exosome protein concentration was determined using BSA linear standard calibration curve. In all experiments, the constant concentration of exosomes, equal to 43 µg of total protein/mL, was held.

### 2.5. Automated Capillary-Based Protein Immunoassay

To confirm successful preparation of exosomes, the cells and EXOs pellet sample were separated using the fully automated capillary-based instrument Jess (ProteinSimple, Bio-Techne, Minneapolis, MN, USA) under reducing (Calnexin) and non-reducing (CD9, CD81) conditions, respectively, using dithiotreitol (DTT) at default manufacturer’s device settings. Total protein from cell culture and exosome fraction was extracted using Mammalian Protein Extraction Reagent (M-PER, Thermo Fisher Scientific, USA) with protease inhibitors and EDTA (Thermo Fisher Scientific, USA). The concentration of the protein samples was determined by Micro BCA Protein Assay Kit as previously described. Protein samples were heated in 70 °C for 5 min (CD9, CD81) and 95 °C for 5 min (Calnexin) and immediately cooled on ice. Samples were loaded in instrument’s plate using 0.7 mg/mL for cell lysates and 0.1 mg/mL for exosome lysate concentrations, and a capillary-based immunoassay was performed. Signal was obtained using chemiluminescence mode using 12–230 kDa Separation Module (Capillary Cartridges SM-W004, ProteinSimple, Bio-Techne, USA) and following antibodies: anti-CD9 (D8O1A) Rabbit Monoclonal Antibody (1:10, Cell Signaling Technology, Danvers, Massachusetts, USA), anti-CD81 (E2K9V) Rabbit Monoclonal Antibody (1:35, Cell Signaling Technology, USA), and anti-Calnexin (C5C9) Rabbit Monoclonal Antibody (1:10, Cell Signaling Technology, USA). Anti-mouse (042-205, ProteinSimple, Bio-Techne, USA) and anti-rabbit (042-206, ProteinSimple, Bio-Techne, USA) secondary antibodies were obtained from Bio-Techne and used undiluted, according to the manufacturer’s manual. Obtained data were analyzed using Compass for SW (ProteinSimple, Bio-Techne, Minneapolis, MN, USA) software version 7.1.

### 2.6. Loading of Doxorubicin (DOX) into Exosomes (EXOs) by Electroporation

Exosomes resuspended in PBS (30 µL, 43 µg protein/mL) were mixed with 570 μL of DOX solutions of different concentrations (0.0575, 0.575 and 1.437 µM) for 15 min at 37 °C. Next, 500 μL of the obtained solution was transferred to the electroporation cuvette. The electroporation was performed in a 0.4 cm gap-width electroporation cuvette using a Gene Pulse Electroporator with a 150 μF capacitor under 350 V [19,43] and then incubated for 30 min at 37 °C to allow for a full recovery of the EXOs’ membrane. The unbound DOX dye was removed by centrifugation twice at 120,000× *g* for 120 min, followed by washing the exosomal pellet with PBS.

The concentration of doxorubicin encapsulated in the exosomes (EXOs@DOX) was determined by measuring the intrinsic fluorescence of doxorubicin after lysis of the EXOs@DOX pellet with 15 µL M-PER lysis buffer (n = 3) (Mammalian Protein Extraction Reagent #78501, Thermo Scientific) using a Fluoroskan Ascent FL (Thermo Scientific) at excitation wavelength *λ*_ex_ = 485 nm and emission wavelength *λ*_em_ = 538 nm.

The DOX loading efficiency in exosomes isolated from SW480 cells was calculated according to the following formula:

$$Loading\;efficiency\;\left(LE\%\right)=\frac{FL\;of\;DOX\;encapsulated\;in\;exosomes}{FL\;of\;DOX\;used}\times 100\%$$

### 2.7. 3,3′-Dioctadecyloxacarbocyanine Perchlorate (DiO) Staining of Exosomes

Exosomes isolated from SW480 cell lines were incubated with 3, 3′ dioctadecyloxa-carbocyanine perchlorate (DiO) lipophilic dye (100 µM stock solution) for 30 min at 37 °C. After incubation, ultracentrifugation at 120,000× *g* for 120 min was performed twice to remove unbound DiO dye. Next, the precipitated pellet of exosomes was gently resuspended with sterile PBS solution.

To quantify intracellular fluorescence levels obtained from DiO-labeled exosomes uptaken by recipient cells (SW480, A549, HBE, CCD 841 CoN), the corrected total cell fluorescence (CTCF) analysis was applied. In this method, to obtain a more accurate fluorescence signal, normalized between the different cell lines, the background fluorescence is subtracted from the total measured fluorescence according to the following formula [57]:

CTCF = Integrated Density − (Area of selected cell × Mean fluorescence of background readings)

### 2.8. Immunofluorescent Staining and Imaging Procedures

For direct immunofluorescence assay, HBE, SW480, A549, and CCD 841 CoN cells were grown for 14 h at 37 °C on microscopic glass coverslips placed in a 35 mm × 10 mm dish with empty SW480-derived exosomes (30 µL, 43 µg protein/mL) or SW480-derived exosomes loaded separately with doxorubicin hydrochloride (final concentration of DOX incorporated in EXOs was 0.10 µM) or DiO-labeled SW480-derived exosomes. The recipient cells’ fixation, staining, and imaging were performed according to the procedure developed earlier [58] with changes. Briefly, after incubation with the exosomes, HBE cells were washed twice in PBS and fixed in 3.7% solution of paraformaldehyde (PFA) for 20 min at room temperature (RT). After fixation, the cells were washed twice with PBS and incubated with 0.5% Tween/PBS solution for 5 min at RT. Then, the sample was again washed twice with PBS and blocked with 3% BSA/PBS solution for 30 min at RT. The cell nuclei were stained with Hoechst 33258/DAPI for 2 min, rinsed with PBS and washed twice with water. Subsequently, the coverslips were mounted on microscope slides using a mounting medium (ProLong™ Gold Antifade Mountant, Invitrogen, Waltham, MA, USA). Slides were examined using FluoView FV10i Confocal Laser Scanning Microscope (Olympus, Tokyo, Japan). Images were captured at 60 × magnification and analyzed using FV10-ASW 3.0 Viewer software after conversion to 24-bit tiff files for visualization. Other software packages, including ImageJ (NIH Image, version 1.53a, USA) and Adobe Photoshop CS6 software (Adobe Systems Incorporated, ver. 13.0, San Jose, CA, USA), were also used.

### 2.9. Metabolic Activity of HBE Cells After Doxorubicin-Loaded Exosomes (EXOs@DOX) Uptake

The metabolic activity of HBE cells after EXOs@DOX uptake was determined using UV-Vis Alamar Blue assay. HBE cells were seeded into 96-well plates at a density of 61,600 cells/well. Then, the cells were cultured at 37 °C for 48 h. Next, the cells were treated with exosomes, doxorubicin encapsulated in the exosomes (final concentration of DOX incorporated in EXOs was 0.10 µM), and doxorubicin solutions (0.10 µM) for 24 h, 48 h, and 72 h. Finally, the Alamar Blue solution was added at a concentration of 0.01%, according to the manufacturer’s manual, and was left to incubate for 4 h at 37 °C. The values of absorbance at 570 and 600 nm were recorded.

## 3. Results

### 3.1. Characterization of Exosomes Isolated from SW480 Cells Using WB, UV-Vis, NTA, and TEM

**Figure 1:**
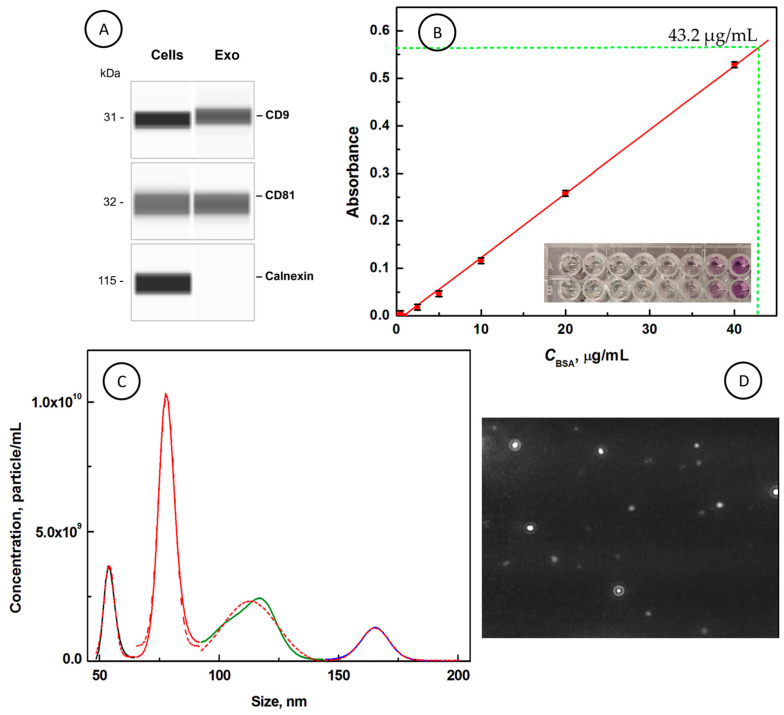
(**A**) Representative lane views from capillary-based immunoassays indicating detection of CD9, CD81, and Calnexin protein expression in the SW480 cell lysate (left panel) and exosomes isolated from SW480 cells (right panel). (**B**) Dependence of the absorbance of BSA solutions at *λ*_abs_ = 570 nm on BSA concentration. INSET: Duplicate absorbance visualization measurements for BSA samples, *C*_BSA_ [µg/mL]: (1) 0, (2) 0.5, (3) 1, (4) 2.5, (5) 5, (6) 10, (7) 20, (8) 40. (**C**) The size distribution of exosomes obtained using the nanoparticle tracking assay (NTA). (**D**) Image from the video of exosomes moving under Brownian motion in the path of a laser beam (parallel to the image) at 488 nm. The raw images are shown in Supplementary Materials.

One of the main goals of this work was to isolate the exosomes from SW480 cell culture media by sequential centrifugation and ultracentrifugation steps and their characterization using molecular biology techniques. First, the expression of an EXO protein marker was corroborated by the Simple Western capillary-based immunoassay. In Figure 1A, clear bands at 31 kDa and 32 kDa are demonstrated, characteristic of CD9 and CD81 tetraspanins, respectively, which belong to the specific exosomal proteins. Cellular contamination was excluded by confirmation of the absence of Calnexin expression in the exosomal fraction. The efficacy of exosomes’ isolation was next assessed based on the total protein content in an isolated intact exosome pellet using a colorimetric bicinchoninic acid (BCA) assay [59,60]. The calibration curve was prepared for bovine serum albumin (BSA) according to the manufacturer’s protocol, and the absorbance signal was read at 570 nm wavelength. In Figure 1B, it is clearly seen that the absorbance intensity increases with a linear range extending from 0 to 40 µg/mL concentration of BSA. In the Inset, the plate with BSA calibration solutions is presented in duplicate. In each experiment, the exosome samples with a constant protein concentration of 43.2 μg/mL were used (total protein content in an exosome pellet was 64.8 µg).

Next, the number of exosomes and their size distribution were determined for a liquid suspension using nanoparticle tracking analysis (NTA). The concentration of EXOs was determined as 2.00 × 10^11^ ± 8.07 × 10^9^ particles/mL. It corresponds to 1.5 × 10^10^ ± 0.61 × 10^9^ exosomes obtained in a pellet. The exosomes showed narrow size distributions with a modal particle diameter of 72.4 ± 4.2 nm and a mean value (size) of 101.6 ± 0.3 nm. In Figure 1C, the distribution of the most common sizes of exosomes is depicted. These sizes have been determined from the plot of *C* (particle/mL) vs. size (nm) and have been estimated using the maximum peak of the Gaussian function. It is shown that the largest area is occupied by exosomes with average sizes centered at 77.9 nm (area 8.1 × 10^10^ (particle/mL) × nm) and 112.9 nm (area 7.0 × 10^10^ (particle/mL) × nm). The exosomes with sizes centered at 54 nm and 165 nm occupied areas of 1.9 × 10^10^ (particle/mL) × nm. Figure 1D presents the image of EXOs moving in a liquid suspension in the path of a beam of scattered light at 488 nm in a chamber sample subjected to NTA. The results obtained confirm the isolation of exosomes from SW480 cells.

**Figure 2:**
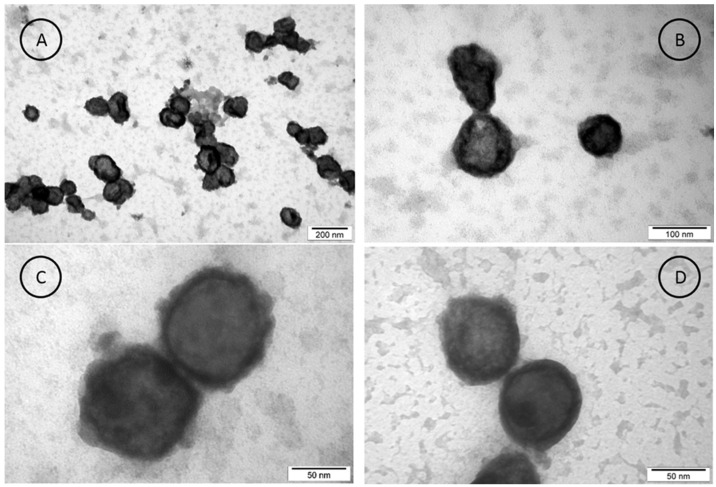
The images of exosomes isolated from SW480 cells obtained using transmission electron microscopy (TEM) at lower (**A**,**B**) and higher (**C**,**D**) resolution.

The size and shape of exosomes isolated from SW480 cancer cells before DOX loading were also supported by transmission electron microscopy (TEM) imaging (Figure 2). It is clearly seen that the extracted EXOs were spherical. The exosomes were heterogeneous in size, showing two main particle classes with average diameters of 69 ± 9 nm and 113 ± 11 nm. The data obtained from the TEM images were consistent with obtained data from NTA particle size distributions.

### 3.2. Loading of EXOs with Anticancer Drug DOX

**Figure 3:**
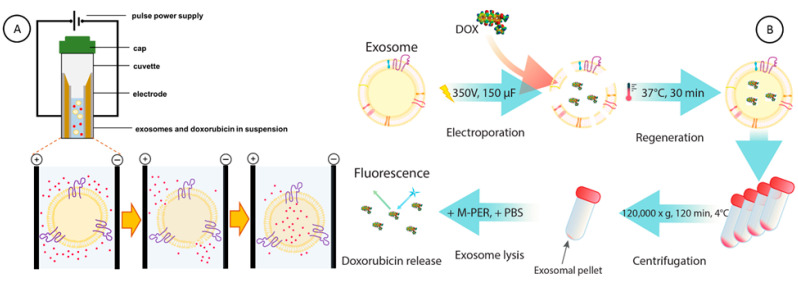
(**A**) Scheme of the electroporation cuvette and loading process of doxorubicin (DOX) into exosomes using the electroporation technique. (**B**) Schematic diagram of DOX incorporation and measurement of encapsulated DOX in exosomes. Figure not in scale.

Next, we investigated the ability of EXOs to entrap and carry doxorubicin hydrochloride (DOX), an anticancer drug, using fluorescence (FL) spectroscopy. In Figure 3A, the scheme of DOX encapsulation in exosomes using the electroporation process is depicted. After the electroporation was carried out at 350 V with a 150 µF discharge capacitor, the exosomes loaded with DOX (EXOs@DOX) were regenerated in a 30 min process at 37 °C and then centrifuged for 2 h at 120,000× *g*. Then, the exosomal pellet with encapsulated DOX was resuspended in PBS with M-PER lysis buffer (PBS/M-PER) (Figure 3B). The fluorescence signal derived from DOX, released from exosomes, was recorded as the analytical signal.

**Figure 4:**
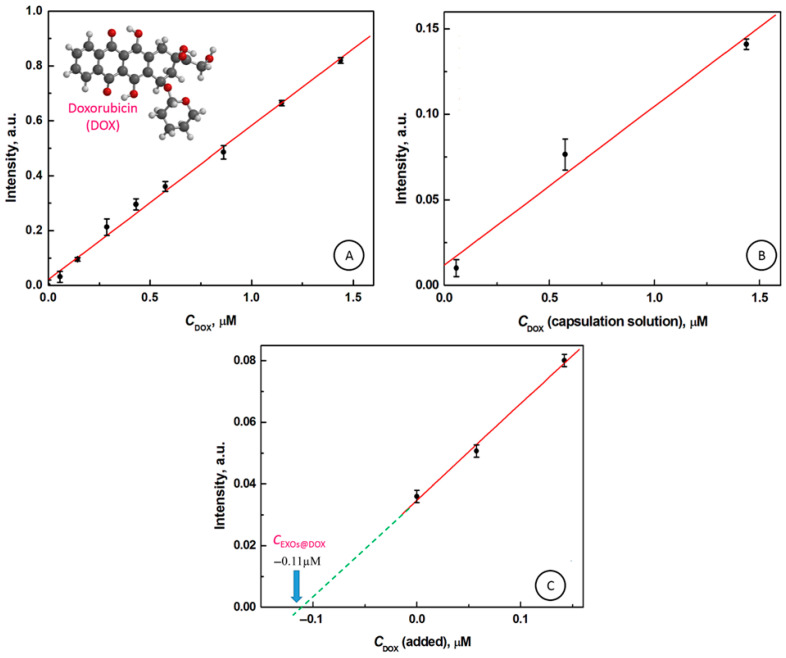
(**A**) Dependence of DOX emission intensity on DOX concentration in M-PER/PBS solution; INSET: the chemical structure of doxorubicin. (**B**) Dependence of the fluorescence intensity of DOX released from exosomes on DOX concentration in a capsulation solution. (**C**) Determination of the concentration of DOX encapsulated in exosomes (*C*_EXOs@DOX_) using the standard addition method; *λ*_ex_ = 485 nm, *λ*_em_ = 538 nm.

In Figure 4A, the calibration curve for doxorubicin fluorescence intensity at 538 nm wavelength, in PBS/M-PER buffer, is presented. The fluorescence intensity *I*_FL,DOX_ increases from 0 to 0.8197 a.u., for concentrations of DOX increasing from 0 to 1.437 µM, respectively. A linear response of the fluorescence intensity was observed in the entire range of DOX concentrations with the correlation coefficient of determination *R*^2^ = 0.997, and parameters of equation *I* = *a* + *bC*_DOX_ are as follows: *a* = 0.0215 ± 0.0117, *b* = 0.5609 ± 0.0162, and *n* = 9. In Figure 4B, the dependence of fluorescence emission of doxorubicin encapsulated in exosomes (EXOs@DOX) on the concentration of DOX in encapsulating solution is presented (n = 3). The concentrations of DOX were determined based on the calibration curve. It is clearly seen that the encapsulation of DOX in EXOs causes a decrease in the fluorescence emission signal of DOX, *I*_FL,EXOs@DOX1_ to 0.010, *I*_FL,EXOs@DOX2_ to 0.076, and *I*_FL,EXOs@DOX3_ to 0.141 a.u. in comparison to the concentration of DOX in the capsulation solution: 0.0575, 0.575, and 1.437 µM, respectively. Next, based on the doxorubicin calibration curve, the concentration of DOX encapsulated in exosomes and the effectiveness of the DOX loading process were evaluated. Using fluorescence spectroscopy, the doxorubicin concentrations in exosomes, equal to 0.10 µM and 0.21 µM, for 0.575 µM and 1.437 µM doxorubicin in capsulation solutions, respectively, were determined. The loading efficiencies (LE) of 17% and 15% for 0.575 µM and 1.437 µM DOX in capsulation solutions, respectively, were achieved.

To corroborate DOX incorporation in exosomes and determine their concentration in exosomes, *C*_EXOs@DOX_, the standard addition method often, used in analytical chemistry to quantify the analyte present in an unknown sample [61,62,63,64,65], was employed. In Figure 4C, the dependence of fluorescence intensity on doxorubicin concentration for lysate of exosomes is presented, with the linear fitting and extrapolation of the line to the intercept with abscissa, DOX incorporated, and two spiked concentrations of DOX, 57 nM and 144 nM (final concentrations), respectively. The absolute value of the negative X-intercept represents the DOX concentration loaded by electroporation into exosomes from 0.575 µM doxorubicin in a capsulation solution. The obtained value of *C*_DOX_ = 0.11 µM. The parameters of the line *I* = a + b*C*_DOX_ are as follows: a = 0.0348 ± 0.0022, b = 0.3133 ± 0.0254, coefficient of determination R^2^ = 0.997, and n = 3. The concentration of DOX found in exosomes lysate using the standard addition method is consistent with the value of doxorubicin concentration found using the fluorescence spectroscopy technique, indicating that the encapsulation process is repetitive.

### 3.3. Encapsulation of DOX Molecules in Exosome Drug Nanocarriers

Exosomes in their structure resemble the unilamellar liposomes which contain the amphiphilic lipid bilayer surrounding an aqueous core [66]. EXOs, similarly, consist of a hydrophobic lipid membrane bilayer and a hydrophilic core; therefore, during the encapsulation process, the drug may be loaded within the lipid bilayer or within the interior dependence of the hydrophobicity/hydrophilicity of drugs. Additionally, during the electroporation process, the external electric field disrupts the exosome membrane, creating a transient state of membrane permeability that allows drug molecules to enter. Since doxorubicin hydrochloride is a hydrophilic drug, we expect DOX molecules to diffuse through the disrupted exosome membrane to the inside of exosomes.

**Figure 5:**
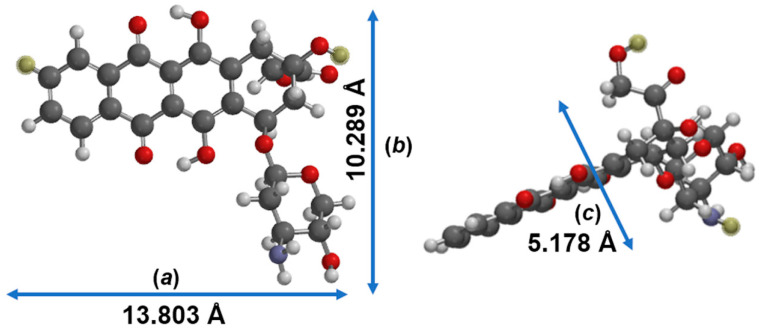
The structure and dimensions of a DOX molecule with the marked length (*a*), width (*b*), and thickness (*c*) determined using Spartan ’14 software (Waveform, Inc. Irvine, CA, USA).

To estimate the maximum number of doxorubicin molecules that can fit in a single exosome drug nanocarrier (Table 1), the chemical structure and dimensions of a DOX molecule were evaluated using Spartan ’14 software (Irvine, CA, USA). The dimensions of the doxorubicin molecule, shown in Figure 5, have been determined as follows: *a* = 13.803 Å, *b* = 10.289 Å, *c* = 5.178 Å, where *a* stands for the DOX molecule length, *b* for DOX width, and *c* for DOX thickness. In the evaluations, the exosomes of two different sizes determined by TEM measurements were used. Assuming the mean value of bilayer thickness as 5.5 nm [67], the inner diameter *d*, surface area *S*, and volume of a single exosome *V*, for smaller exosomes, were calculated as *d* = 58 nm, *S* = 1.056 × 10^−14^ m^2^, and *V* = 1.021 × 10^−22^ m^3^ and for larger exosomes as *d* = 102 nm, *S* = 3.277 × 10^−14^ m^2^, and *V* = 5.554 × 10^−22^ m^3^. The comparison of experimental values for DOX molecules loaded into exosomes with theoretical data is presented in Table 1.

### 3.4. Uptake of EXOs@DOX into HBE Cells

The most common sites of CRC metastases are the liver and the lungs. It is known that at the time of diagnosis of colorectal cancer, up to 20% of patients already experience metastatic disease [68], and pulmonary metastases occur in 15% of metastatic CRC patients [69]. Several studies have also found that 6–8% of colon carcinomas and 10–18% of rectal carcinomas metastasize to the lung [70].

**Figure 6:**
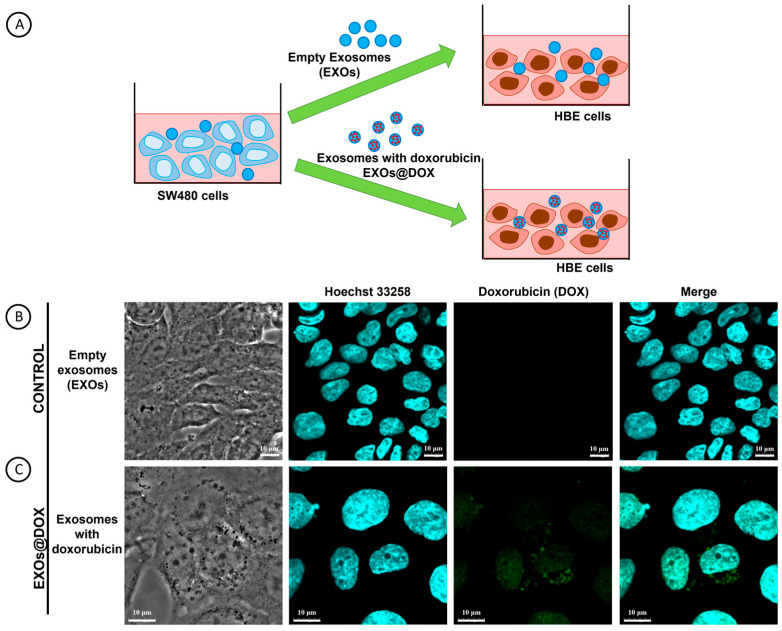
(**A**) Schematic diagram of the experimental procedure (not in scale). (**B**,**C**) Immunofluorescence images of HBE cells incubated for 14 h using confocal laser microscopy with (**B**) empty and (**C**) doxorubicin-loaded SW480-derived exosomes; (**B**) control, cells treated with empty exosomes; (**C**) visualization of doxorubicin (green fluorescence). Nuclei are stained with Hoechst 33258 (blue fluorescence). Objective magnification × 60, bars = 10 μm.

Therefore, in the next step, investigations of the uptake of exosomes into the human bronchial epithelial (HBE) cells found at the lung environment interface, and their effect on the cells metabolic function, were performed using confocal laser microscopy. The HBE cells reflect the normal epithelial lining of the lung airways, making them a biologically valuable model for studying the early changes induced in lungs before the secondary tumor’s formation. These cells can respond to signals released by tumors, such as cytokines and exosomes, changing their secreted factors, extracellular matrix components, and inflammatory responses which are characteristic of the premetastatic lung environment. Furthermore, HBE cell cultures are easier to control and manipulate, allowing for consistent and reproducible analysis of early events associated with metastasis [71,72]. In Figure 6A, the scheme of the experiment is depicted. The HBE cells were treated with naϊve exosomes, as well as doxorubicin-loaded exosomes (EXOs@DOX) isolated from SW480 for 14 h. The final concentration of DOX incorporated into EXOs was 0.10 µM. We found that the HBE cells incubated with empty exosomes, delivered from SW480 cells, show no fluorescence (Figure 6B), whilst the HBE cells incubated with exosomes loaded with doxorubicin emit green fluorescence related to encapsulated DOX (Figure 6C). The merge panel shows the presence of doxorubicin in cell nuclei stained blue with Hoechst 33258 (blue fluorescence) and their close vicinity in the perinuclear regions of the cytoplasm. It indicates that the exosomes effectively entered the target cells and delivered the drug.

**Figure 7:**
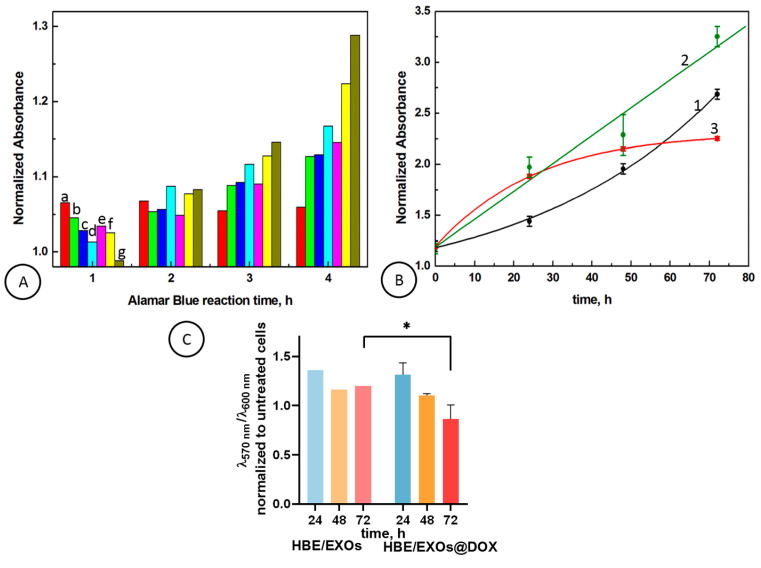
(**A**) The normalized absorbance (570 nm vs. 600 nm) of Alamar Blue assay after different times of interaction for different amount the cells in wells; [cells/well]: (a—red) 8800, (b—green) 17,600, (c—dark blue) 25,500, (d—cyan) 34,000, (e—magenta) 42,500, (f—yellow) 52,800, (g—olive) 61,600. (**B**) The normalized absorbance of Alamar Blue assay for HBE cells (1) treated with empty exosomes (2) and exosomes loaded with doxorubicin EXOs@DOX (3). (**C**) The absorbance ratio normalized to untreated HBE cells for HBE cells treated with empty exosomes (EXO) and exosomes loaded with doxorubicin (EXOs@DOX). Statistical analysis was performed in GraphPadPrism 8 (GraphPad Inc., San Diego, CA, USA) using a two-way ANOVA followed by a multiple comparisons test. *p*-values shown represent * *p* < 0.05.

Next, the viability of HBE cells after EXOs@DOX uptake using Alamar Blue assay was evaluated. In this assay, resazurin is reduced to resorufin by the living cells and their metabolic activity. Hence, the absorbance was read at 570 nm using 600 nm as the reference wavelength, providing the analytical signal. In Figure 7A, the optimalization of assay conditions including time of Alamar Blue reaction and number of cells in wells is presented. It is clearly shown that the number of 8800 cells per well (bar 1) is not enough to reduce resazurin to resorufin. This is indicated by the fact that the ratio of absorbance at 570 nm to absorbance at 600 nm decreases during the assay reaction time from 1 to 4 h. The higher number of cells in wells including 17,600 (bar 2), 25,500 (bar 3), 34,000 (bar 4), 42,500 (bar 5), 52,800 (bar 6), and 61,600 (bar 7) shows the sufficient amounts of cells to reduce the Alamar Blue solution. The ratio of absorbance at 570 nm to absorbance at 600 nm increases for 1, 2, 3, and 4 h of interaction of cells with Alamar Blue reagent. The results indicate the growth of the cells that maintain the reducing environment and their metabolic activity. Therefore, in further experiments, the Alamar Blue interaction time of 4h was chosen, and the amount of 61,600 cells per well was used. Line 1 in Figure 7B depicts the dependence of normalized absorbance of HBE cells after 24, 48, and 72 h. It is observed that the normalized absorbance increases in an exponential way, suggesting that the cells grow and proliferate over the culture period with sufficient surface. In Figure 7B, Lines 2 and 3, the effects of the addition of empty exosomes and DOX-loaded exosomes on the normalized absorbance of HBE cells are presented. The presence of empty exosomes in the HBE cell culture, isolated from SW480 cells, causes an increase in the absorbance ratio in a linear fashion (Line 2). It indicates that the exosomes improve proliferation of HBE cells.

After the uptake of exosomes loaded with doxorubicin (EXOs@DOX) into HBE cell culture, it is seen that the metabolic activity and viability of the growing HBE cells are decreasing (Figure 7B, Line 3) and the normalized absorbance reaches a plateau after 50 h. This suggests that the doxorubicin delivered by exosome enters the cells, causing the inhibition of cell growth due to the diminishing of the reducing environment of the cells.

In Figure 7C, the absorbance ratio at 570 and 600 nm, normalized to untreated HBE cells, is performed for HBE cells treated with empty exosomes and exosomes loaded with doxorubicin (0.1 µM). It is shown that the absorbance ratio of Alamar Blue reagent for HBE cells treated with doxorubicin encapsulated in SW480-derived exosomes has decreased by about 16% and 36% after 48 h and 72 h, respectively, whereas the empty exosomes caused decreases of about 15% and 12% after 48 h and 72 h.

The obtained results confirm our early findings that the increase in the cells’ proliferation due to the empty exosomes and doxorubicin present in EXOs@DOX cause the inhibition of cell growth. It would be necessary to perform additional specific tests to verify the apoptotic potential of doxorubicin delivered by exosomes, but the obtained results indicate good application potential of exosomes extracted from SW480 cells as an efficient platform for the doxorubicin loading and delivery system, as well as the stimulators of the modulation of metabolic activity of HBE cells. We found that the free doxorubicin (0.1 µM) caused a decrease in the normalized absorbance about 3% after 72 h. It indicates that doxorubicin encapsulated in exosomes enters the cells more effectively as compared to the free drug.

### 3.5. Uptake of EXOs@DiO by Recipient Cells

**Figure 8:**
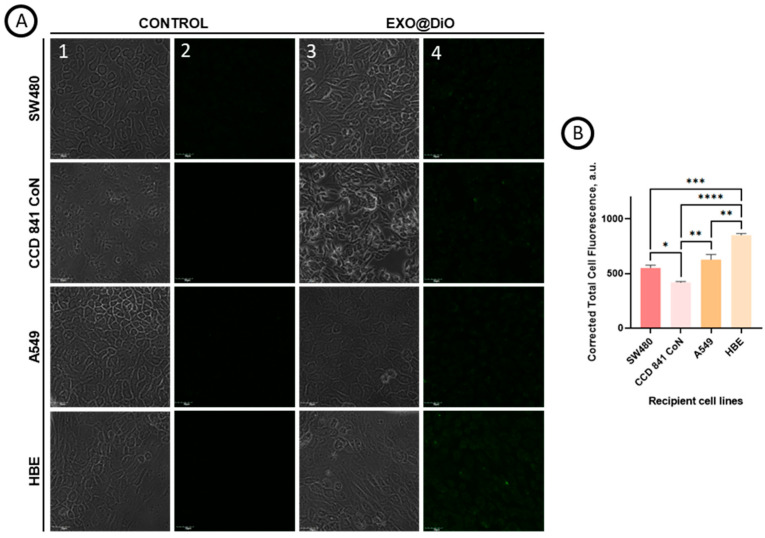
(**A**) The cellular uptake of DiO-labeled SW480 derived exosomes in parental (SW480) cells and recipient (HBE, A549, CCD841 CoN) cells after 14 h incubation. Confocal laser microscopy bright field (column 1,3) and fluorescence (column 2,4). (**B**) The calculated corrected total cell fluorescence (CTCF) obtained from Fiji (ImageJ) software version 2.14.0/1.54r. Statistical analysis was performed in GraphPad Prism 8 (GraphPad Inc.) using Shapiro–Wilk normality test and one-way ANOVA, followed by Tukey’s post hoc multiple comparisons test. CTCF data are presented as mean ± SEM. *p*-values shown represent * *p* < 0.05; ** *p* < 0.01, *** *p* < 0.001, **** *p* < 0.0001. Objective magnification × 60, bars = 30 μm.

To investigate whether exosomes derived from SW480 cells show tropism toward parental and other cells, we labeled exosomes with DiO fluorescent dye (EXOs@DiO) and tracked their uptake by the recipient SW480, A549, CCD 841 CoN, and HBE cells using confocal laser microscopy. Figure 8A presents the intracellular visualization and internalization of exosomes without (column 1,2) and with (column 3,4) lipophilic dye DiO accumulated in the exosomal lipid bilayer. The results show a significant green fluorescence intensity in cells treated with EXOs@DiO after 14 h of incubation, indicating the cellular exosomes’ uptake (Figure 8A, column 4). In Figure 8B, the corrected total cell fluorescence (CTCF) for different recipient cell lines is presented. It is clearly seen that the CTCF signal for HBE cells (851.2 a.u.) was higher than for SW480 (550.5 a.u.), A549 (627.5 a.u.), and CCD 841 CoN (418.3 a.u.) cells (Figure 8B). Our study showed that SW480-derived exosomes exhibit a tropism toward their origin SW480 cells and a pronounced tropism toward HBE and A549 cells, whereas their uptake by CCD 841 CoN cells is comparatively low, suggesting limited interaction.

## 4. Discussion

A similar drug loading efficiency was obtained by Li et al. [73]. The authors determined the drug loading in exosomes by HPLC method with an efficacy of 7.2% for PTX and 11.7% for DOX. Wei et al. [37] obtained DOX encapsulation efficiency in exosomes isolated from bone marrow mesenchymal stem cells (BM-MSCs) of approximately 12% when the mass ratio of EXO/DOX was 93:7. Thakur et al. [74] showed that the loading efficiency of DOX into SF7761 stem-cell-like and U251-GMs-derived-exosomes were 19.7% and 7.86%, respectively. The authors performed exosome loading with saponin as the cellular membrane permeabilizing agent, using a microfluidic device at the injection flow rate of 50 μL/min. The doxorubicin loading content in exosomes secreted by mesenchymal stem cells (MSCs) and exosomes tagged with 5TR aptamer against MUC1 was 17 ± 5.1% and 21 ± 3.1%, respectively [75]. The utilization of the other systems for the effective delivery of doxorubicin to tumors such as a metal–organic framework (MOF)-coated MnO_2_ nanosheet results in a DOX loading capacity of 9.1% [76]. Loading doxorubicin into MSC-derived exosomes modified with carboxylated Fe_3_O_4_ nanoparticles resulted in approximately 38% encapsulation efficiency based on volumetric quantification [77]. It is worth noting that exosomes secreted from cells are already natural carriers for numerous biomolecules such as proteins and nucleic acids; hence, some cargo capacity for drug loading is expected [18,73].

In order to improve the targeting feasibility of exosomes and increase the DOX loading efficiency, the modification and engineering of the exosome surface have been recently proposed. Tian et al. [78] utilized in their research the exosomes produced by immature dendritic cells (imDCs), engineered to express lysosome-associated membrane glycoprotein 2b (Lamp2b) fused with iRGD (CRGDKGPDC) targeting peptide for αv integrin. The efficiency of DOX loading to chemically conjugated exosomes with two types of phenylboronic acids, i.e., 3-aminophenylboronic acid (APBA) and 4-carboxyphenylboronic acid (CPBA), was 36.7% [79]. The enhancement of doxorubicin content in exosomes can also be obtained by the conjugation of the therapeutic agent with a 1,2-distearoyl-*sn-glycero*-3-phosphoethanolamine (DSPE) lipid chain (L-DOX). The loading efficiency of L-DOX adsorbed on the surface of exosomes was close to 20%, three times greater than free doxorubicin [80]. Another strategy designed to enhance DOX’s selectivity, uptake, and therapeutic effectiveness is the drug delivery system based on fusion of exosomes with liposomes. It was recently shown that the encapsulation percentage of doxorubicin in the pegylated pH-sensitive liposome fused with exosomes (HF-pHSL/EV-DOX) was 88.9 ± 2.4%. [81].

The promising potential of exosomes as targeted drug delivery systems is also attributed to their cell/tissue-specific tropism, which facilitates selective uptake by target cells. Numerous studies clearly show a direct link between the exosomal content including glycoproteins, tetraspanins, integrins or immunoglobulins and a tissue-specific tropism of exosomes [82]. Furthermore, it has been shown that altering the protein composition, including knock-down or integrins silencing, can affect tropism and the efficiency of internalization. For example, exosomes enriched in integrin α6 in complex with β1 and β4 subunits show a preferential tropism toward fibroblasts and epithelial cells in lungs while exosomal integrin αvβ5 was linked to liver metastasis. It was also found that the organo-tropic tumor-derived exosomes prime distant microenvironments (pre-metastatic niches) in a manner that is sufficiently potent to support metastasis, even by tumor cells that inherently have low competence for colonizing in those organs [83]. Limongi et al. [82] reported that exosomes originating from lymphocytes preferentially internalize into the parental cells. It was also demonstrated that the similarity in surface proteins and lipids between exosomes and their parental cells promotes preferential uptake by the same cells. Emam et al. [84] have also found that the exosomes isolated from murine adenocarcinoma C26 cells (C26-Exos) were more readily internalized by the same donor C26 cells in comparison with the allogeneic murine melanoma B16BL6 cells. The higher uptake and internalization of exosomes collected from B16BL6 cells was observed in donor B16BL6 cells than in allogeneic C26 cells [85]. Moreover, Feng et al. [86] showed that exosomes are taken up more efficiently by phagocytic cells than non-phagocytic cells. The results obtained by Smyth et al. [87] indicate instead that exosomes derived from the MCF-7, MDA-MB-231, and PC3 cancer cell lines are not preferentially associated with the parental cell lines. Similar results were obtained by Horibe et al. [88]. The authors showed that exosomes released by donor cells are taken up in a non-selective manner by target cells, and exosome uptake is not identical across all cell types.

Our results are consistent with those reported previously. The observed higher internalization of exosomes by HBE and A549 cells, reflecting the protein and lipid profile of respiratory cells, suggests a strong potential of EXO@DOX for future investigations into pre-metastatic niche attenuation. The uptake of exosomes into parental cell line instead shows the potential use of SW480-EXO for effective treatment of the same cancer cell type. Therefore, our results have offered valuable insights into the efficiency of DOX loading into SW480-derived exosomes and the innate preferential uptake of these exosomes by cells.

The findings obtained after exosome and DOX-loaded exosome uptake in HBE cells also correspond to results obtained by other researchers. Ji et al. [89] showed that exosomes derived from SW480 and SW620 cells promote the proliferation of mouse endothelial 2F2B cells. Janowska-Wieczorek et al. [90] found that platelet-derived microvesicles (PMV) and exosomes strongly stimulated the proliferation/survival of human lung cancer A549 cells. The exosomes isolated from colon cancer cell lines, HT-29, SW480, and LoVo, were found to increase the cellular proliferation of HT-29 cells in an investigation performed by Kim et al. [91]. Yasodha et al. [92] reported that exosomes, derived from human metastatic colorectal cancer cell line SW620, increase primary colorectal cell line HCT116 proliferation. This occurs since the exosomes carry various factors, including growth and angiogenesis-related factors [93] as well as oncogenes [94]. Schindler et al. [38] proved that exosomal doxorubicin was taken up faster into the recipient cells than free DOX or liposomal doxorubicin. Moreover, the authors showed that after 4 h, approx. 15 times more free doxorubicin is needed to elicit an equivalent fluorescent signal after incubation of HEK293 cells compared to doxorubicin-loaded exosomes. The enhanced cellular uptake efficiency of exosome-loaded doxorubicin in comparison with free DOX in the osteosarcoma MG63 cell line was also shown by Wei et al. [37]. Kim et al. [79] observed the enhancement of MDA-MB-231 breast cancer cell cytotoxicity after interaction with DOX-loaded PBA-conjugated exosomes in comparison with cells treated with free and DOX-loaded exosomes in non-conjugated exosomes, respectively.

However, the findings of this study should be evaluated with certain limitations. First, the heterogeneity of EXOs size and variability in the loading capacity of specific exosomal fractions should be taken into consideration to evaluate which of them is primarily responsible for the observed metabolic effects. Normak et al. found that the exogenous loading of extracellular vesicles results in a substantial variability [95]. The overall efficiency of GFP protein uptake remained low, particularly for small fractions of extracellular vesicles.

In addition to differences in drug-loading efficiency, it is worth noting that the timing and mechanism of cellular entry may vary with vesicle size.

Moreover, our research was conducted using HBE cell model, which, although a well-researched representative of the pulmonary interface, does not reflect the complexity of the lung microenvironment. Therefore, it requires further in-depth studies encompassing the complexity of interactions with target cells, the selectivity and efficiency of delivery, the safety profile, and the utility of the carriers, including the efficiency of intracellular drug release as well as additional models, e.g., co-culture with immune or stromal cells. Consequently, an undertaken approach remains in the realms of proof-of-concept rather than a thoroughly investigated solution to challenges associated with cancer therapy.

## 5. Conclusions

The obtained results indicate that SW480 cells constitute a high-quality exosome source, which can be examined using molecular biology methods, nanoparticle tracking analysis, and TEM imaging. EXOs also serve as an excellent delivery system for the anticancer drug doxorubicin (DOX), encapsulated during electroporation process, achieving 15–17% of loading efficiency. The procedure can be successfully monitored by fluorescence spectroscopy and the standard addition method and analytical chemistry approach. The prepared exosomal nanocarriers enabled the efficient delivery of DOX into human bronchial epithelial (HBE) cells, verified through confocal laser scanning microscopy. Owing to the intrinsic affinity of metastatic colon cancer cells toward airway-interface cells, this finding is of particular importance for the future efforts on suppressing colon-to-lung metastatic spread; nevertheless, additional investigations are required. The performed Alamar Blue assay showed that the metabolic activity of HBE cells can be modulated by the DOX-loaded exosomal nanocarriers, causing the inhibition of cell growth and diminishing the cells’ reducing environment by about 16% and 36% after 48 h and 72 h of interaction with EXOs@DOX. Preferential uptake of the investigated exosomes was demonstrated using DiO-labeled SW480-derived exosomes. Normalized DiO fluorescence signal was assessed through image analysis and calculation of total corrected cell fluorescence across different cell lines, revealing cell-line-dependent variations in exosomal internalization. Human bronchial epithelial (HBE) cells, serving as a model for a potential pre-metastatic niche for circulating colon cancer cells, showed the strongest EXO@DiO uptake signal. A relatively high exosomal affinity was noted for lung carcinoma A549, along with parental SW480 colon cancer cells, whereas healthy colon epithelial cells CCD 841 CoN exhibited significantly lower levels. The results corroborate the key role of exosome parental cell line in predicting exosomal internalization preferences, potentially guiding the development of enhanced targeted drug-delivery strategies using biocompatible exosomal nanocarriers in targeted tumor treatment approaches—a growing area of interest in personalized medicine.

## Figures and Tables

**Table 1:** The estimation of the maximum number of DOX molecules that can fit in a single exosome drug nanocarrier.

**Theoretical Number of DOX Molecules Entrapped Inside EXOs**		
Concentration of DOX in encapsulated solution	DOX molecules per exosome (smaller)	DOX molecules per exosome (bigger)
1.437 µM	8.84 × 10^4^	4.81 × 10^5^
0.575 µM	3.54 × 10^4^	1.92 × 10^5^
**Experimental Number of DOX Molecules Entrapped Inside EXOs**		
Concentration of DOX entrapped in EXOs	DOX molecules per exosome (smaller)	DOX molecules per exosome (bigger)
0.21 µM	1.29 × 10^4^	7.02 × 10^4^
0.1 µM	6.15 × 10^3^	3.34 × 10^4^

## Data Availability

The data used to support the findings of this study are included in the article.

## Supplementary Materials

The following supporting information can be downloaded at: https://www.mdpi.com/article/10.3390/cancers18030379/s1, raw images.

## Abbreviations

The following abbreviations are used in this manuscript:

CRC	colorectal cancer
EXOs	exosomes
SW480	human colon cancer cell line
HBE	human bronchial epithelial cell line
A549	human lung carcinoma cell line
CCD 841 CoN	human healthy epithelial colon cell line
EMT	epithelial-to-mesenchymal transition
NTA	nanoparticle tracking analysis
TEM	transmission electron microscopy
DOX	doxorubicin
DiO	3,3′-Dioctadecyloxacarbocyanine perchlorate
DAPI	4′,6-Diamidine-2′-phenylindole dihydrochloride
EXOs@DOX	doxorubicin-loaded exosomes, exosomes loaded with doxorubicin
FBS	fetal bovine serum
DMEM	Dulbecco’s modified Eagle Medium High Glucose
PBS	Phosphate-buffered saline
PMV	platelet-derived microvesicles
BM-MSCs	bone marrow mesenchymal stem cells
