# Supplementary figures and images for: Spectroscopic Studies of the Modulation of Metabolic Activity of HBE Cells by Doxorubicin Hydrochloride-Loaded SW480-Derived Exosomal Nanocarriers
Source: Cancers (Basel). 2026 Jan 26;18(3):379. doi: 10.3390/cancers18030379 (PMC12896753; doi:10.3390/cancers18030379)

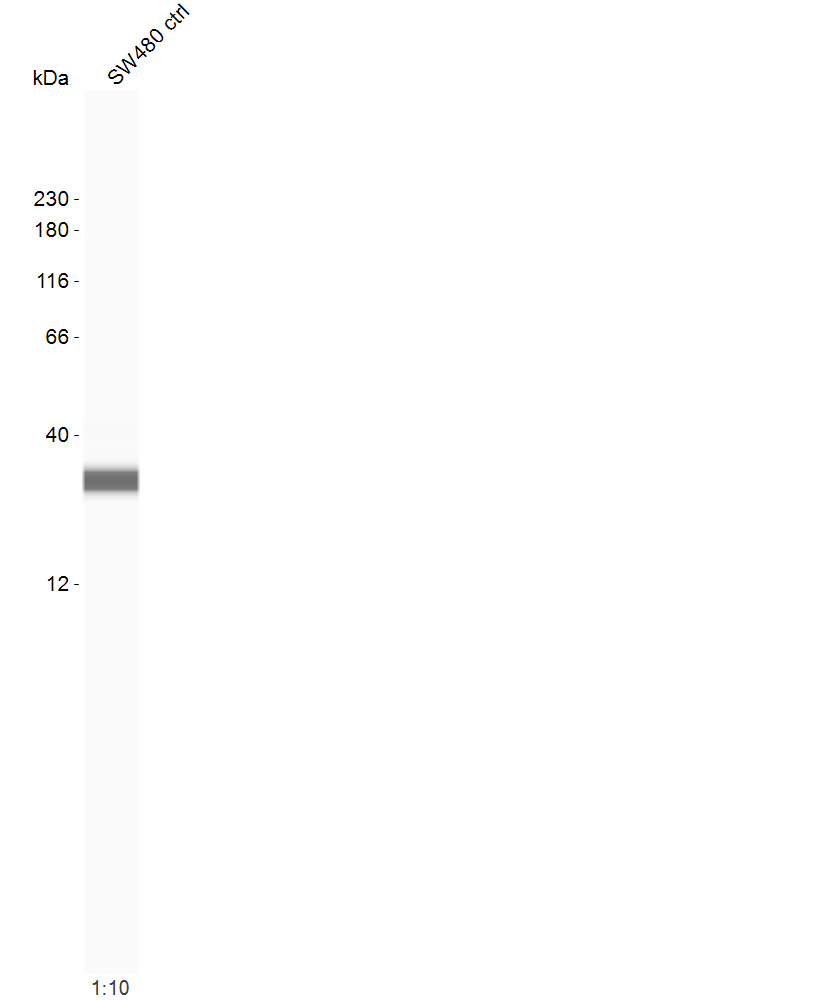

Supplement: Supplementary file 1 [file cancers-18-00379-s001.zip › Blots/sw480 lysate cd81 raw 70C 5 min non reducing.png]

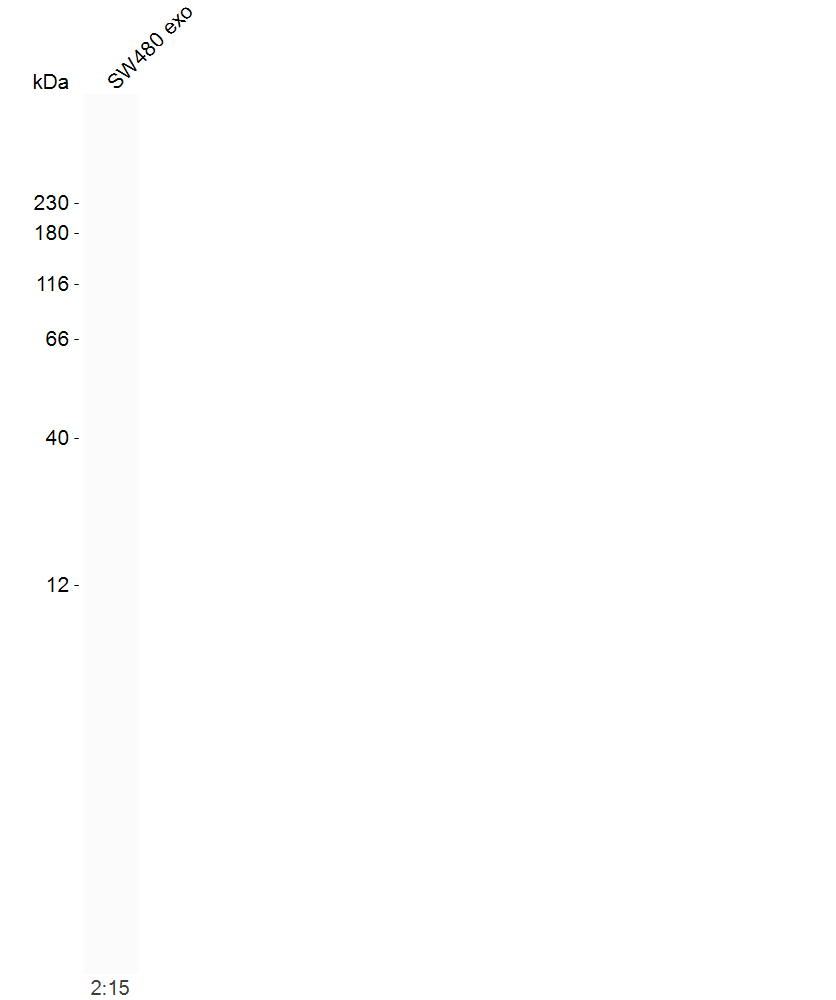

Supplement: Supplementary file 1 [file cancers-18-00379-s001.zip › Blots/sw480 exo calnexin raw 95C 5 min DTT.png]

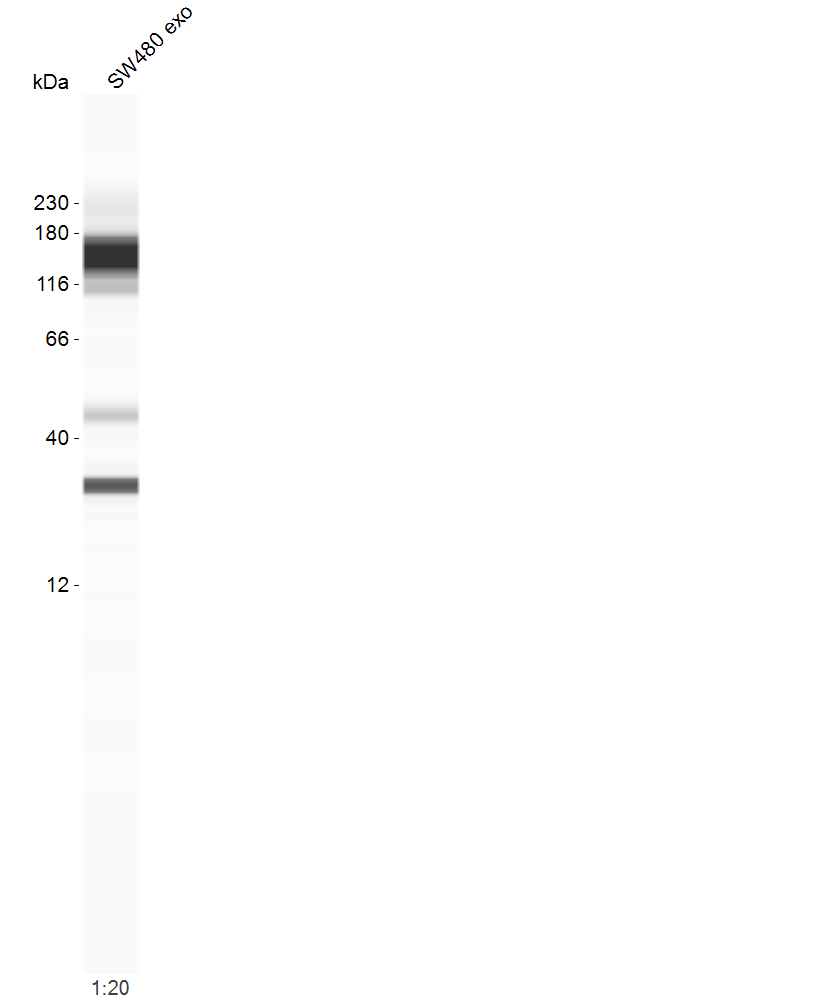

Supplement: Supplementary file 1 [file cancers-18-00379-s001.zip › Blots/sw480 exo cd9 raw 70C 5 min non reducing.png]

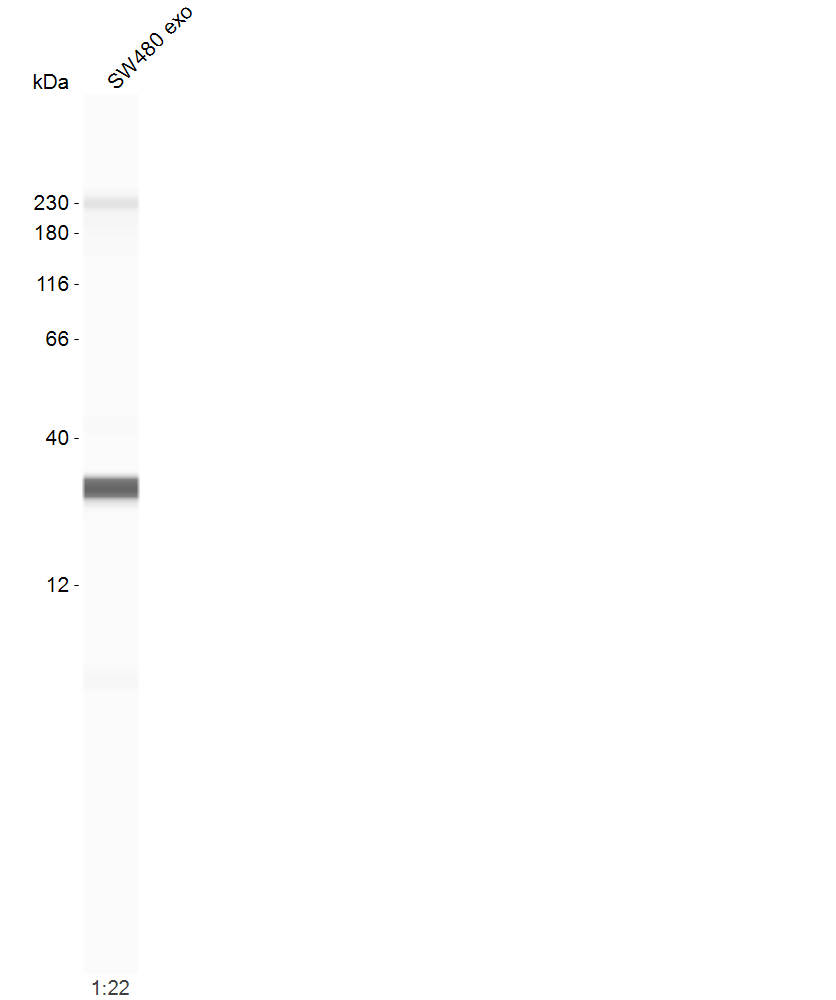

Supplement: Supplementary file 1 [file cancers-18-00379-s001.zip › Blots/sw480 exo cd81 raw 70C 5 min non reducing.png]

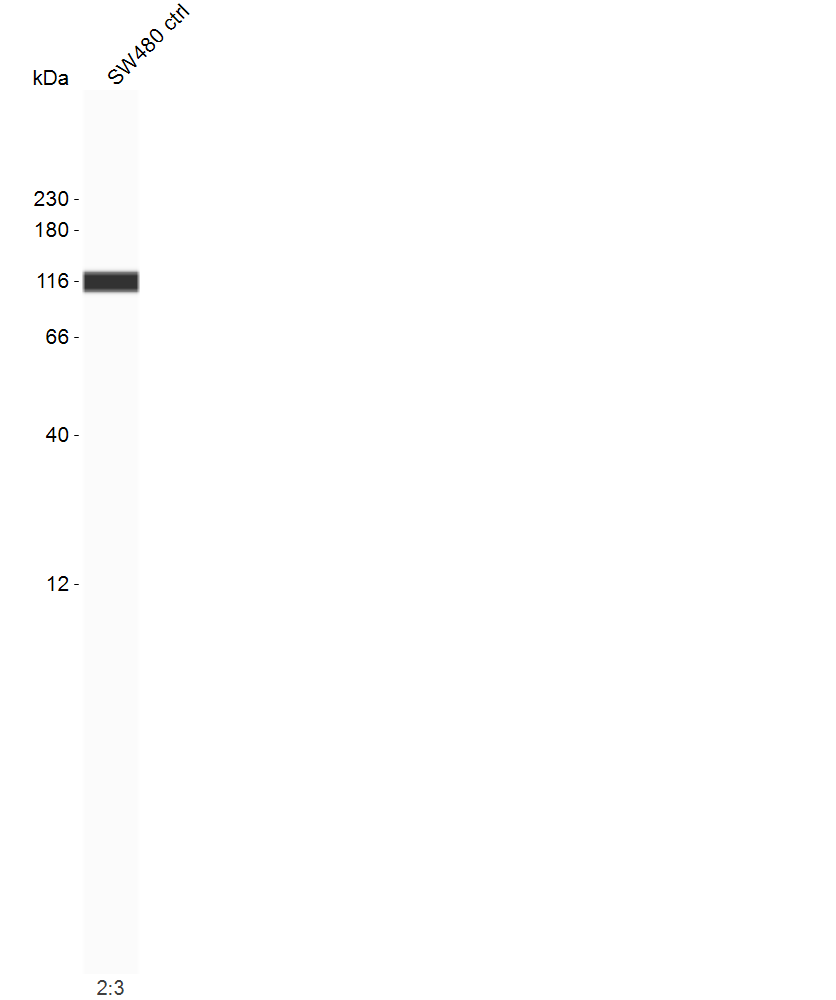

Supplement: Supplementary file 1 [file cancers-18-00379-s001.zip › Blots/sw480 lysate calnexin raw 95C 5 min DTT.png]

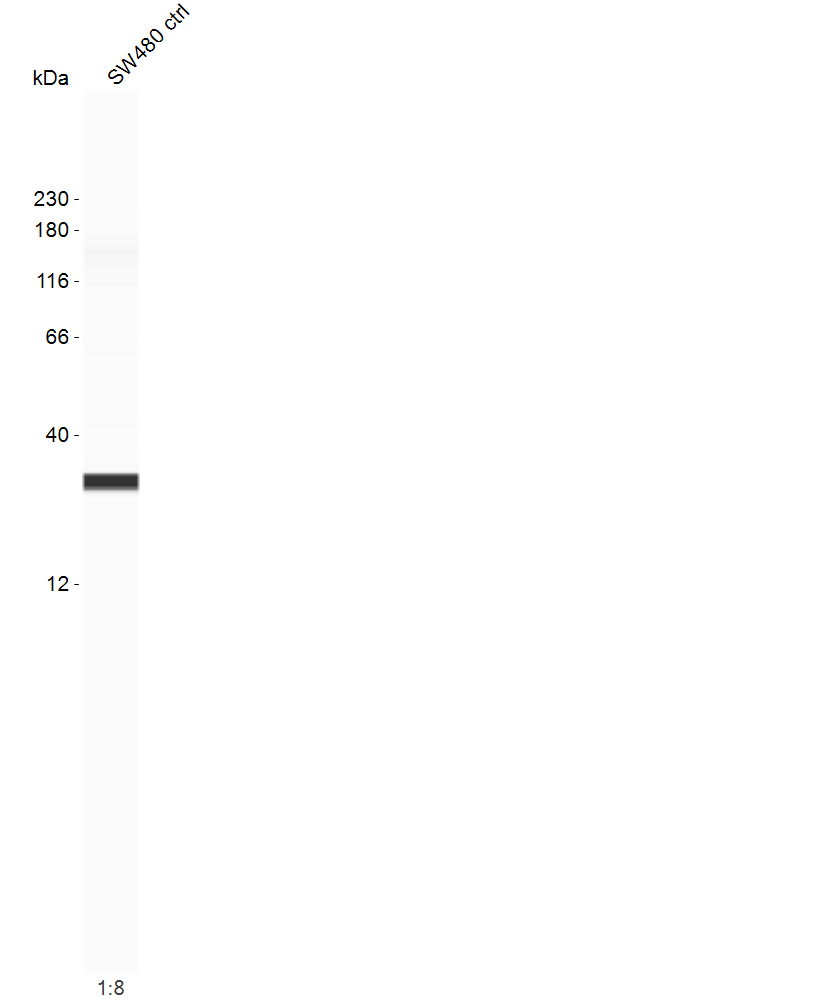

Supplement: Supplementary file 1 [file cancers-18-00379-s001.zip › Blots/sw480 lysate cd9 raw 31 kda 70C 5 min non reducing.png]
